# Textpresso Central: a customizable platform for searching, text mining, viewing, and curating biomedical literature

**DOI:** 10.1186/s12859-018-2103-8

**Published:** 2018-03-09

**Authors:** H.-M. Müller, K. M. Van Auken, Y. Li, P. W. Sternberg

**Affiliations:** Division of Biology and Biological Engineering, California Institute of Technology, Pasadena, CA 91125 USA

**Keywords:** Literature curation, Text mining, Information retrieval, Information extraction, Literature search engine, Ontology, Model organism databases

## Abstract

**Background:**

The biomedical literature continues to grow at a rapid pace, making the challenge of knowledge retrieval and extraction ever greater. Tools that provide a means to search and mine the full text of literature thus represent an important way by which the efficiency of these processes can be improved.

**Results:**

We describe the next generation of the Textpresso information retrieval system, Textpresso Central (TPC). TPC builds on the strengths of the original system by expanding the full text corpus to include the PubMed Central Open Access Subset (PMC OA), as well as the WormBase *C. elegans* bibliography. In addition, TPC allows users to create a customized corpus by uploading and processing documents of their choosing. TPC is UIMA compliant, to facilitate compatibility with external processing modules, and takes advantage of Lucene indexing and search technology for efficient handling of millions of full text documents.

Like Textpresso, TPC searches can be performed using keywords and/or categories (semantically related groups of terms), but to provide better context for interpreting and validating queries, search results may now be viewed as highlighted passages in the context of full text. To facilitate biocuration efforts, TPC also allows users to select text spans from the full text and annotate them, create customized curation forms for any data type, and send resulting annotations to external curation databases. As an example of such a curation form, we describe integration of TPC with the Noctua curation tool developed by the Gene Ontology (GO) Consortium.

**Conclusion:**

Textpresso Central is an online literature search and curation platform that enables biocurators and biomedical researchers to search and mine the full text of literature by integrating keyword and category searches with viewing search results in the context of the full text. It also allows users to create customized curation interfaces, use those interfaces to make annotations linked to supporting evidence statements, and then send those annotations to any database in the world.

Textpresso Central URL: http://www.textpresso.org/tpc

## Background

Biomedical researchers face a tremendous challenge in the vast amount of literature, an estimated 1.2 million articles per year (as a simple PubMed query reveals), that makes it increasingly difficult to stay informed. To aid knowledge discovery, information from the biomedical literature is increasingly captured in structured formats in biological databases [1], but this typically requires expert curation to turn natural language to structured data, a labor-intensive task whose sustainability is often debated [2–5]. Moreover, database models cannot always capture the richness of scientific information, and in some cases, experimental details crucial for reproducibility can only be found in the references used as evidence for the structured data. Thus, because of the overwhelming number of publications and data, needs have shifted towards information extraction.

Biocuration is the process of “extracting and organizing” published biomedical research results, often using controlled vocabularies and ontologies to “enable powerful queries and biological database interoperability” [6]. Although the details of curation for different databases may vary, to accomplish these goals biocuration involves, in general, three essential tasks: 1) identification of papers to curate (triage); 2) classification of the relevant types of information contained in the paper (data type indexing); and 3) fact extraction, including entity and relationship recognition (database population) [7–10].

As the number of research articles increases, however, it becomes very challenging for biocurators to efficiently perform these three tasks without some assistance from natural language processing and text mining. To address this challenge, we developed an automated information extraction system, Textpresso [11, 12], to efficiently mine the full text of journal articles for biological information. Textpresso split the full text of research articles into individual sentences and then labeled terms in each sentence with tags. These tags were organized into categories, groups of words and phrases that share semantically meaningful properties. In turn, the categories were formally organized and defined in a shallow ontology (i.e., organized in a hierarchy), and served the purpose of increasing the precision of a query.

Textpresso full text searches could be performed in three ways: 1) by entering words or phrases into a search field much like popular search engines; 2) by selecting one or more categories from cascading menus; or 3) by combining keyword(s) and categories. Search results were presented to users as lists of individual sentences that could be sorted according to relevance (subscore-sorted) or their position within the document (order-sorted). Using the full text of *C. elegans* research papers, we demonstrated the increased accuracy of searching text using a combination of categories from the Textpresso ontology and words or phrases [12]. In addition, because they identify groups of semantically meaningful terms, categories can be used for information extraction in a semi-automated manner (i.e. search results are presented to biocurators for validation), thus speeding up, and helping to improve sustainability of, curation tasks in literature-based information resources, such as the Model Organism Databases (MODs) [7, 13]. Textpresso’s full text search capabilities have been used by a number of MODs and data type-specific literature curation pipelines, e.g., WormBase [7, 13], BioGrid [14], FEED [15], FlyBase [16] and TAIR [17]. The utility of semi-automated curation has been demonstrated as well by other groups who have incorporated semi-automated text mining methods into their curation workflows [18–21].

Nonetheless, we sought to improve upon the Textpresso system to better respond to the needs of biocurators and the text mining community. Much effort has been devoted to understanding the critical needs of the biocuration workflow. Through community-wide endeavors such as BioCreative (Critical Assessment of Information Extraction in Biology), the biocuration and text mining communities have come together to determine the ways in which text mining tools can assist in the curation process [7–10, 22–25]. Using the results of these collaborations, as well as our own experiences with biocuration at WormBase and the Gene Ontology (GO) Consortium, we identified areas for further Textpresso development (see Table 1 for a comparison of the old and new Textpresso system). Specifically, for biocurators, we have greatly increased the size of the full text corpus by including the PubMed Central Open Access (PMC OA) corpus and adding functionality that allows users to upload papers to create custom literature sets for processing and analysis. In addition, sentences matching search criteria may now be viewed within the context of the full text allowing for easier validation of text mining outputs. Further, TPC allows biocurators to create customized curation forms to capture annotations and supporting evidence sentences, and to export annotations to any external database. This new feature eases the incorporation of text mining results into existing workflows. For software developers, we have implemented a modular system, wherein features can be reused as efficiently as possible, with minimal redundancy in effort required for support of different databases and types of curation. The TPC system is based on the Unstructured Information Management Architecture (UIMA) which makes it possible to employ 3rd-party text mining modules that comply with this standard. Lastly, for both biocurators and the text mining community, we have implemented feedback mechanisms whereby curators can validate search results to improve text mining and natural language processing algorithms. Below, we describe the development of the Textpresso Central system, the key features of its user interface, and a curation example demonstrating integration of Textpresso Central with Noctua, a curation tool developed by the GO Consortium [26].

**Table 1:** Comparison between the old Textpresso system and Textpresso Central

Feature	Textpresso	Textpresso Central
Full text searching	✔	✔
PMC OA corpus		✔
Custom corpus creation		✔
Literature subdivision		✔
Keyword and category searching	✔	✔
Search by paper section	✔	
Keyword exclusion, search filters	✔	✔
Category browsing and searching	✔	✔
Sort by relevance or year	✔	✔
Search results viewed within context of full text		✔
Highlight and annotate full text		✔
Customizable annotation interface		✔
Communication with external curation databases		✔
UIMA compliant		✔

## Implementations

### Unstructured information management architecture

The Unstructured Information Management Architecture (UIMA) has been developed by IBM [27] and is currently an open source project at the Apache Software Foundation [28] to support the development and deployment of unstructured information management applications that analyze large volumes of unstructured information, such as free text, in order to discover, organize and deliver relevant knowledge to the end user. The fundamental data structure in UIMA is the Common Analysis Structure (CAS). It contains the original data (such as raw text) and a set of so-called “standoff annotations.” Standoff annotations are annotations where the underlying original data are kept unchanged in the analysis, and the results of the analysis are appended as annotations to the CAS (with references to their positions in the original data). UIMA allows for the composition of complicated workflows of processing units, in which each of the units add annotations to the original subject of analysis. Thus, it supports well the composition of NLP pipelines by allowing users to reuse and customize specific modules. This is also the basic idea behind U-Compare (http://u-compare.org/), an automated workflow construction tool that allows analysis, comparison and evaluation of workflow results [29].

UIMA is well suited for our purposes as we seek compatibility with outside processing modules. Our plan to combine several NLP tools and allow curators to assemble them via a toolbox according to their needs is nicely accomplished via U-Compare. The various, diverse needs of curators can more readily be met when pipelines can easily be modified and modules swapped in and out, allowing curators to design and experiment as they wish. UIMA allows for convenient application of in-house and external modules as the framework is used widely in the NLP community. Modules can be easily integrated into Textpresso Central, for example, the U-Compare sentence detectors, tokenizers, Part-of-Speech (POS)-taggers and lemmatizers. Their semantic tools such as the Named Entity Recognizers (NERs) (see http://u-compare.org/components/components-semantic_tools.html) are well known in the NLP community, and since they are all UIMA compliant, can easily be integrated into Textpresso Central. Thus, overall compatibility of Textpresso Central with software and databases of the outside world will improve.

The implementation and incorporation of UIMA in the system is straightforward. We use the C++ version available from the Apache Software Foundation website which makes processing fast (we can process up to 100 article per minute on a single processor). Implementing UIMA into Textpresso Central takes several days for one developer, but this is a one-time cost.

### Software package used

Besides UIMA, Textpresso Central features state-of-the-art software libraries and technologies, such as Lucene [30, 31] and Wt, a C++ Web Toolkit [32]. Lucene provides the indexing and search technology needed for handling millions of full text papers; Wt delivers a fast C++ library for developing web applications and resembles patterns of desktop graphical user interface (GUI) development tailored to the web. With the help of these libraries and their associated concepts we designed a system with the features as follows.

### Types of annotations

The structure of the CAS file in the UIMA system builds on standoff annotations to the original subject of analysis (SofA) string. All derived information about the SofA are stored in this way, and Textpresso Central annotations work the same way. Our system will know three different kind of annotations:

#### Lexical annotations

These are annotations based on lexica or dictionaries. Each lexicon is associated with a category, and categories can be related through parent-child relationships. All categories and the terms in their respective lexicon are stored in a Postgres database. A UIMA annotator analyzes the SofA string of a CAS file and appends all found lexical annotations to the CAS file.

#### Manual annotations

All annotations created manually through a paper viewer and curation interface are first stored in a Postgres database. A periodically run application will analyze the table and append these annotations to the CAS file, so they can be displayed in the paper viewer for further analysis by the curation community as well as TM and Machine Learning (ML) algorithms. Lucene indexes these annotations and makes them searchable.

#### Computational annotations

The system has the capability to incorporate various machine learning algorithms such as Support Vector Machines (SVMs), Conditional Random Fields (CRFs), Hidden Markov Models (HMMs) and third party NERs to classify papers and sentences, recognize biological entities, and extract facts from full text. The results of these computations are stored as annotations in the CAS file as well. Besides computational annotations provided by the Textpresso Central system by default, users will be able to run algorithms on sets of papers they select in the future, and store and index their annotations.

### Basic processing pipelines

**Fig. 1:**
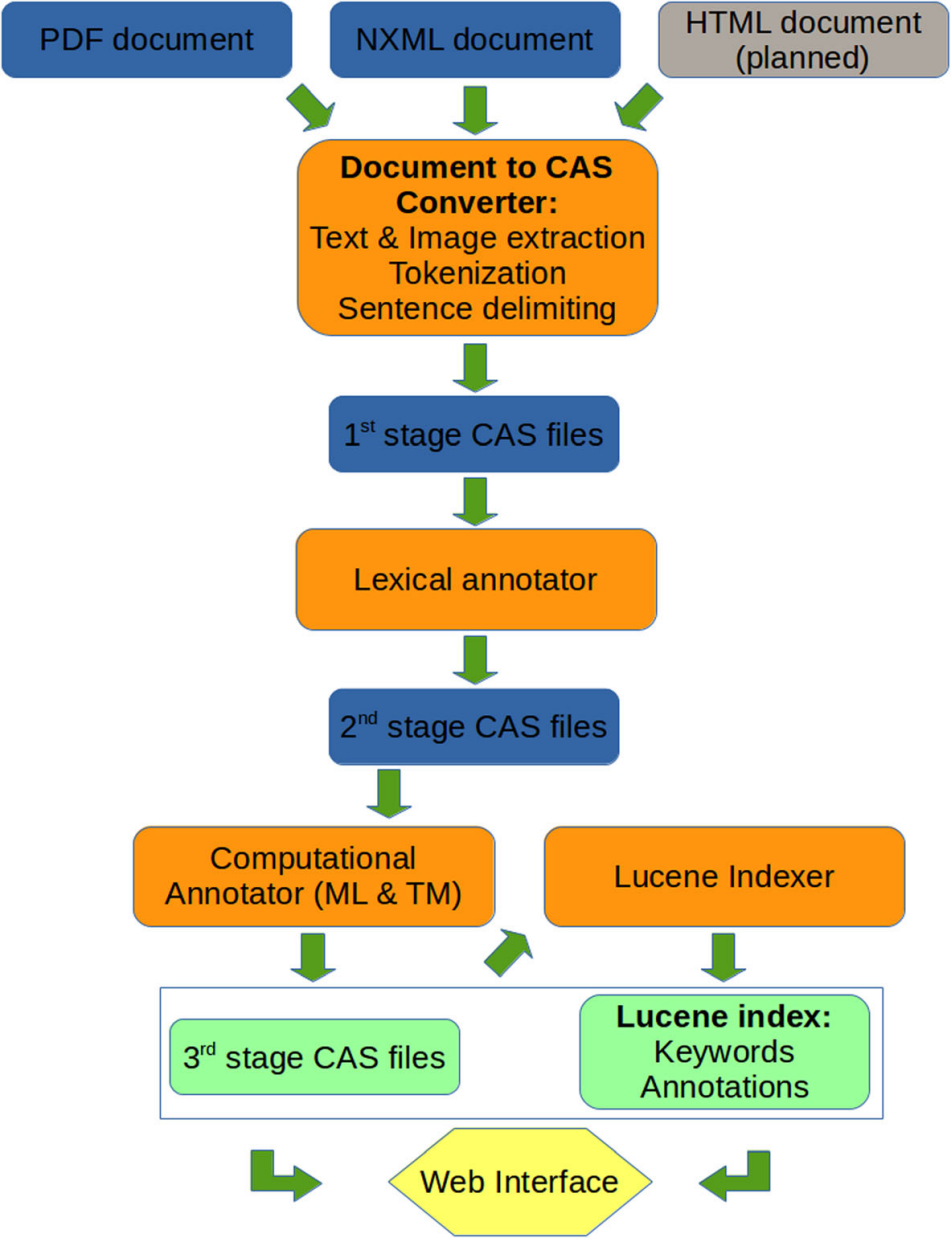
Basic processing pipelines for the Textpresso Central system. The processing includes the full text as well as bibliographic information

Each research article in the Textpresso corpus undergoes a series of processing steps to be readied for the front-end system. In addition, processed files will be available for machine learning and text mining algorithms. Figure 1 illustrates the following steps.

- A *converter* takes the original file, tokenizes it, forms a full text string containing the whole article (SofA, see above), and identifies word, sentence, paragraph, and image information which is written out as an annotation into a file which we call a 1st-stage CAS file. Currently, there are two formats that we can parse for conversion, NXML (for format explanation, see section “Literature Database” below) and PDF. We have written programs for their conversion in C++ which make processing files fast (on average a second for PDFs and a fraction of a second for NXML on a single processor core).
- The *lexical annotator* reads in the CAS file produced by the converter and loads lexica and categories from a Postgres table to find lexical entries in the SofA. It labels each occurrence in the SofA with the corresponding category name and annotates the position in it. These annotations are written out into a 2nd-stage CAS file. Once again, our own implementation in C++ combined with a fast internal data structure to hold the (admittedly large) lexicon (tree) produces annotations on the order of a second per article (single processor core).
- The *computational annotator* will run the 2nd-stage CAS file through a series of default machine learning and text mining algorithms such as NERs. The resulting annotations will be added to the CAS file and written out as a 3rd-stage CAS file.
- The *indexer* indexes all keywords and annotations of the 3rd-stage CAS file and adds it to the Lucene index for fast searching on the web. We are using the C++ implementation that the Apache Foundation is offering for Lucene, resulting in an index rate of around 30 articles per minute and processor core.

### Literature database

The Textpresso Central corpus is currently built from two types of source files: PDFs and NXMLs. The NXML format is the preferred XML tagging style of PubMed Central for journal article submission and archiving [33]. Corpora built from PDFs are more restrictive in nature, i.e. access restrictions will be enforced according to subscription privileges. For NXMLs, we currently use the PMC OA subset [34], which we plan to download and update monthly. To subdivide the Textpresso Central corpus into several sub-corpora that can be searched independently and aids in focusing searches on specific areas of biology, we apply appropriate regular expression filtering of the title, journal name, or subject fields in the NXML file. For example, for the sub-corpus ‘PMCOA Genetics’ we filter all titles, subjects, and journal names for the regular expression ‘[Gg]enet’. Similar patterns apply to all other sub-corpora. This method is only a first attempt to generate meaningful corpora as it has its shortcoming; keywords in title, subject lines and journal names might not be sufficient to classify a paper correctly. Therefore it will be superseded with more sophisticated methods (see Future Work in the Conclusion section).

### Categories

There are two types of categories in Textpresso Central. One type is made from general, publicly well-known ontologies such as the Gene Ontology (GO) [26, 35], the Sequence Ontology (SO) [36, 37], Chemical Entities of Biological Interest (ChEBI) [38, 39], the Phenotype and Trait Ontology (PATO) [40, 41], Uberon [42, 43], and the Protein Ontology (PRO) [44, 45]. In addition, Textpresso Central contains organism-specific ontologies, such as the *C. elegans* Cell and Anatomy and Life Stage ontologies [46]. We periodically update these ontologies, which can be downloaded in the form of an Open Biomedical Ontology (OBO) file, and process and convert them into categories for Textpresso Central. These files include synonyms for each term, and we include them in our system too. For text mining purposes, however, formal ontologies are not necessarily ideal, as natural language used in research articles does not always overlap well with ontology term names or even synonyms. Therefore, we include a second type of category composed of customized lists of terms (and their synonyms). These lists are usually meant for use by a group of people such as MOD curators, who would submit them to us for processing. They are transformed into OBO files and then enter the same processing pipeline as the formal ontologies. They can be accessed by anyone on the system, in contrast to user-uploaded categories that only a particular user has access to. The latter will be implemented in the near future. The customized categories are typically listed under the type of curation for which they were generated, e.g., Gene Ontology Curation or WormBase Curation.

For selection on the website, categories are organized into a shallow hierarchy with a maximum depth of four nodes. This organization allows users to take some advantage of parent-child relationships in the ontologies, without necessarily having to navigate the entire ontology within Textpresso Central. If specific ontology terms are required for searches, those terms can be entered into the search box in the Pick Categories pop-up window and added to the category list (see below).

### Web Interface and modules

**Fig. 2:**
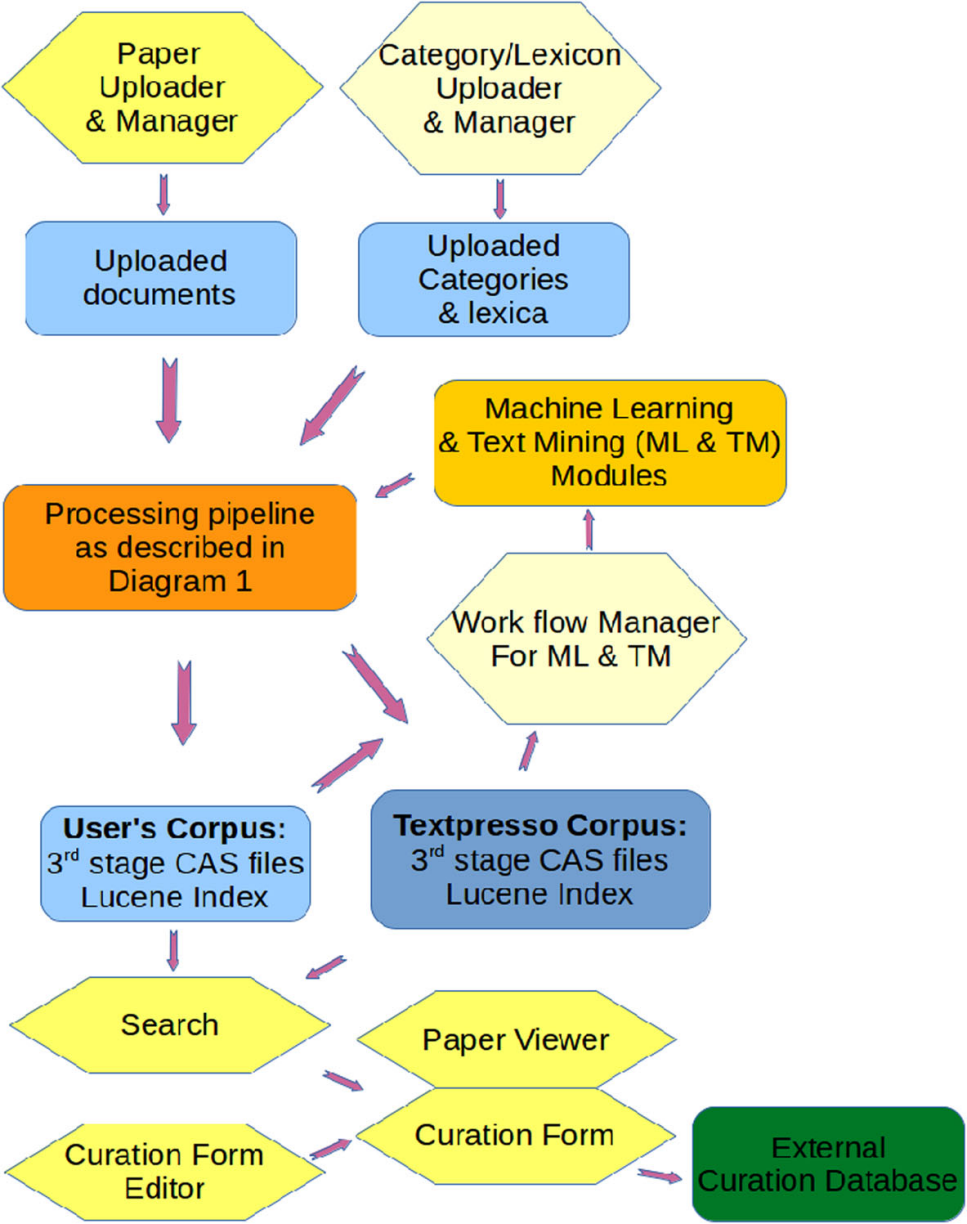
Components of the web interface (hexagons) and their interactions with data and processing units of the system (rectangles). The bright yellow components have been implemented, the light yellow ones are planned

We have designed the new interfaces based on our extended experience with the old Textpresso system as well as feedback from WormBase curators, utilizing a GitHub tracker, who have tested the new system while it was being developed. Figure 2 shows how the web interface interacts with processing modules (shown in yellow in the figure and designated by italics in subsequent text) and the back-end data of the system. The Lucene index and correspondingly all 3rd-stage CAS files of the Textpresso Central corpus are available for the web interface used by the curator. Documents uploaded by the user through the *Papers Manager are* processed in the same way as the Textpresso Central corpus.

The user should first create a username and password. The *Login* system is used to enter user information and define groups and sharing privileges with other people and groups. All customization features and annotation protocols described below require a login so data and preferences can be stored.

**Fig. 3:**
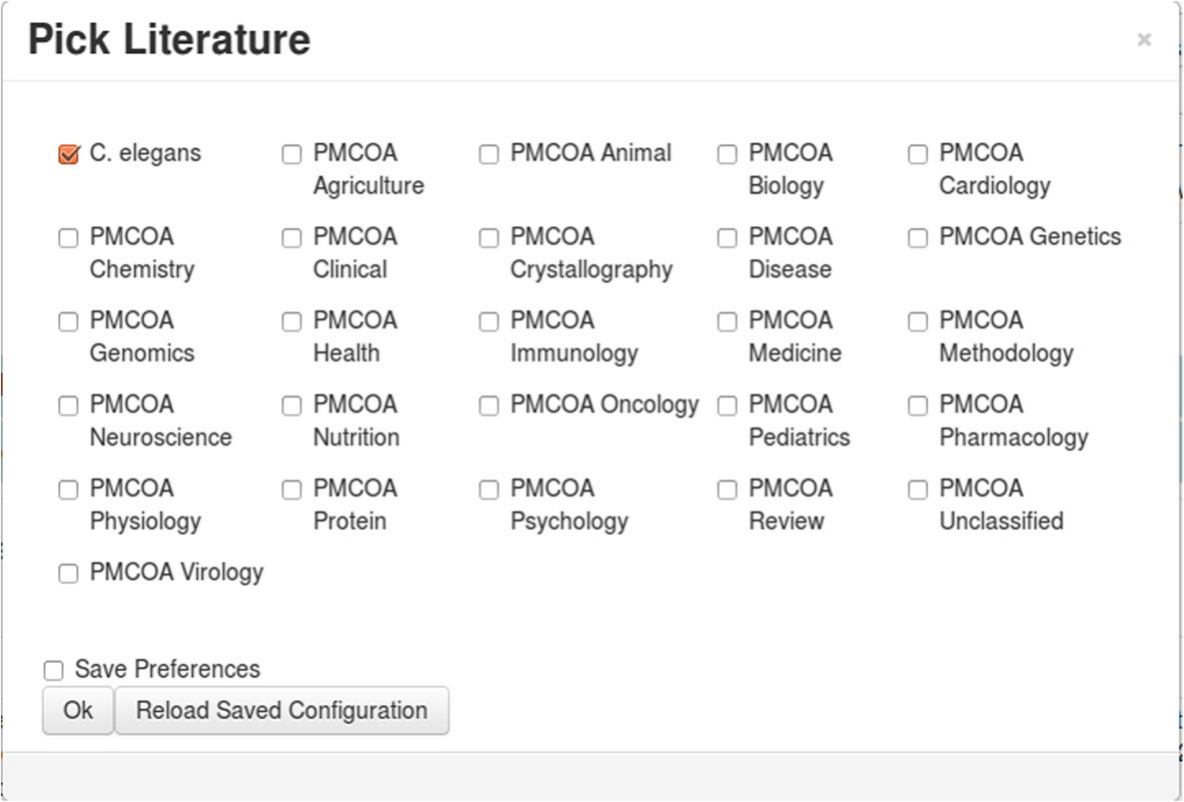
Searches can be restricted to particular literatures

The *Search* module (described in more detail in the Results section) allows for searching the literature for keywords, lexical (category), computational, and manual annotations. It is based on Lucene and uses its standard analyzer (see [47] for more details on analysis). Search results are usually sorted by score which is calculated by Lucene via industry-known term-frequency*inverse-document-frequency (tf*idf) scoring algorithms and then normalized with respect to the highest scoring document (other ranking-score schemes will be offered in the future). As an alternative to score, search results may also be sorted by year. Several common-use filters such as author, journal, year, or accession, as well as keyword exclusion, are available to refine search results. As in the original Textpresso system, search scope can be confined to either sentence or document level. Furthermore, searches can be restricted to predefined sub-literatures (Fig. 3) as described above.

Papers listed in the search results can be selected for viewing in the *Curation* module. In this module a selected paper can be loaded into the paper viewer which allows the curator to read the full paper including.jpg, .png and .gif figures (the display of other figures formats such as ppm will be available in future releases). The curator can also scroll through highlighted matching search results, and view all annotations made to that paper. Keyword and category search capabilities within the paper are also available. The curator can select arbitrary text spans that can be used to fill a fully-configurable web-based curation form, and make manual annotations with it. Once the curation form is filled and approved by the curator, he or she can submit it to an external database in Javascript object notation (JSON) format or a parametrized Uniform Resource Identifier (URI). The curation case study described in the Results section including Fig. 10 shows more detail about this module.

**Fig. 4:**
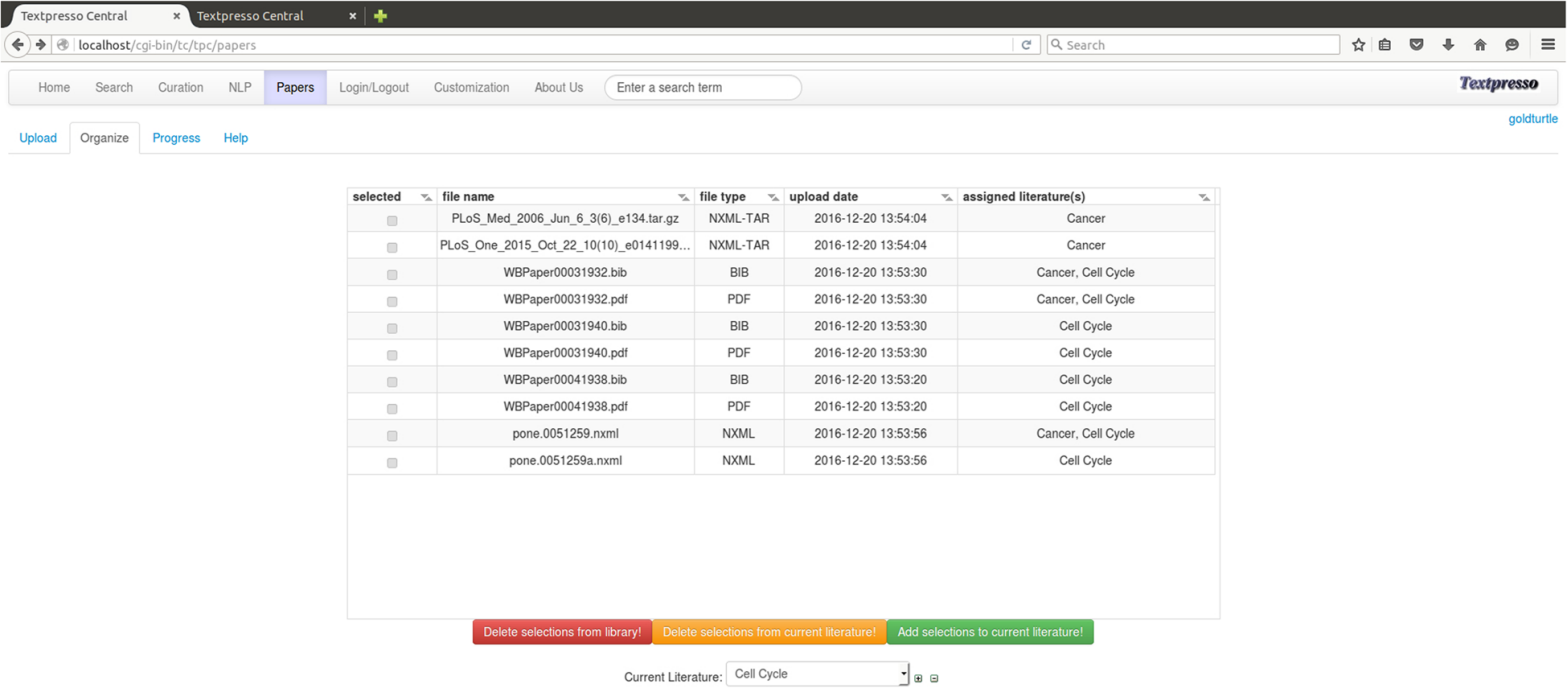
The paper manager. Papers can be uploaded in NXML or PDF format and then organized into literatures as shown here

In addition to the Textpresso Central corpora provided by us, users can upload small sets (on the order of 100 s) of papers in the *Papers* module. Texpresso Central currently accepts papers in PDF and NXML format, and once uploaded, the user can organize them into different literatures (Fig. 4). Automatic background jobs on the server tokenize them, perform lexical annotations, index them, and then make them available online. These background jobs process 100 papers within a few minutes, so the user can work with her own corpus almost immediately.

**Fig. 5:**
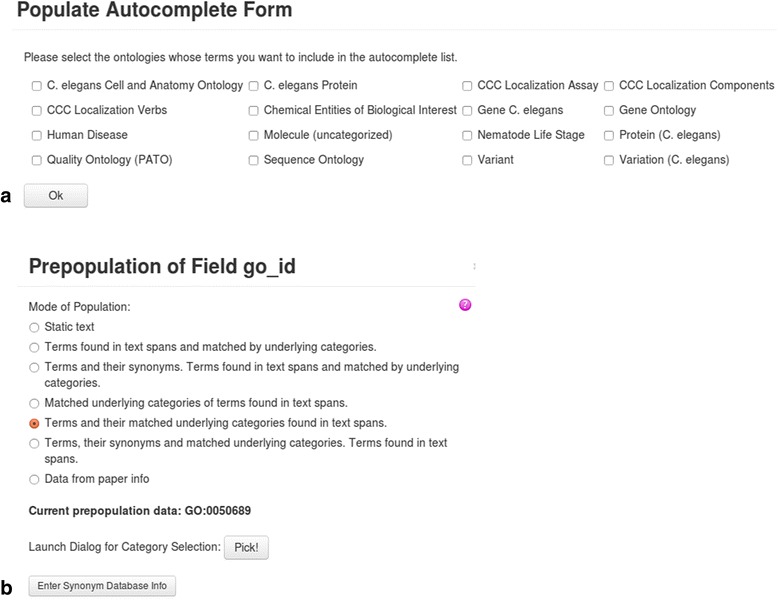
**a** Columns of Postgres tables can provide auto-complete and validation information and are specified in this interface. **b** Fields can be prepopulated in various ways, among them with terms and underlying categories found in text spans that are marked by the curator

The *Customization* module allows users to adjust the settings of many aspects of the site, such as selecting the literature to be searched and creating the curation form. The interface for creating curation forms enables the user to specify an unlimited number of curation fields and the type of each entry field, such as line edit, text area, pull-down menu, or check box. Fields can be placed arbitrarily on a grid and named. Each entry field features auto-complete functionality and can be constrained by a validator. Both auto-complete and validator can be defined through columns in Postgres tables, external web services that can be retrieved from anywhere on the Internet, or the categories present in Textpresso Central. To enhance curation efficiency, fields can be pre-populated with static text, bibliographic information from the paper, or specific terms and/or category entries found in the highlighted text spans, along with their corresponding unique identifiers, if applicable (Fig. 5). Other parameters such as the form name, and the URL to which a completed form should be posted can be defined as well.

## Results

### Textpresso central searches

Like the original Textpresso, Textpresso Central allows for diverse modes of searching the literature, from simple keyword searches to well-defined, targeted searches that seek to answer specific biological questions. In addition, Textpresso Central employs several different types of search filters that allow users to restrict their searches to a subset of the available literature, as well as an option to sort chronologically to always place the most recent papers at the top of the results list. In all cases, TPC searches the full text of the entire corpus. Examples that illustrate Textpresso Central search capabilities are discussed below.

1. Keyword searches
  A simple keyword search can be deployed from the Textpresso central homepage from the *Search* module that can be reached by clicking on the ‘advanced search’ link next to the keyword search box on the homepage or from the ‘search’ link in the tabbed list at the top of the page. In keyword searches multiple words or phrases can be combined according to the specifications of the Lucene query language e.g. use of Boolean operators (AND OR) placing phrases in quotation marks (“DNA binding”) or grouping queries with parentheses.

**Fig. 6:**
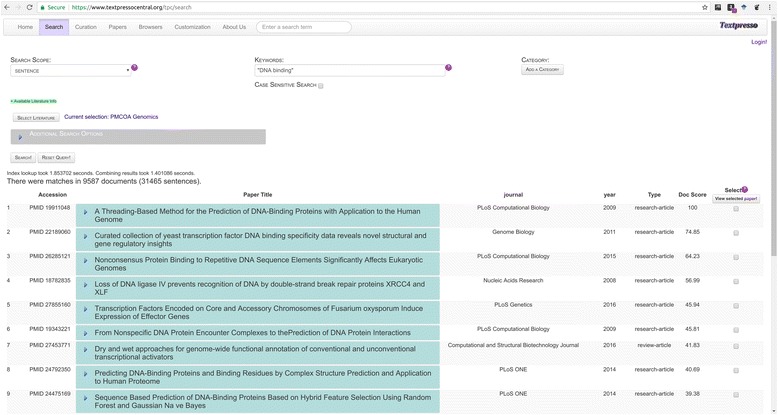
Textpresso Central keyword search

Figure 6 illustrates the results of a keyword search of the PMC OA Genomics sub-corpus for the exact matches to the phrase “DNA binding”. This search returns 31,465 sentences containing the phrase “DNA binding” in 9587 documents, sorted according to relevance (Doc Score) (search performed on 2017–11-17). Search results initially display the paper Accession, typically the PubMed identifier (PMID), Paper Title, Journal, Year, Paper Type, and Doc Score. To view matching sentences and their individual search scores, users can click on the blue arrowhead next to the paper title. The resulting display will show the sentences with matching terms color-coded, bibliographic information for the paper (Author, Journal, Year, Textpresso Literature sub-corpus and Full Accession), and the option to view the paper abstract.

As described, multiple keywords or phrases can be combined in a search according to the specifications of the Lucene query language. Thus, if the user wished to specifically search for references to DNA binding and enhancers, perhaps to find specific gene products that bind enhancer elements, they could modify the above search to: “DNA binding” AND enhancer. In addition, setting the search scope to require search terms be found together in a sentence, and not just in the whole document, enhances the chances of finding more relevant facts in the search results.

2) Category searches
  From its inception, one of the key features of the Textpresso system has been the ability to search the full text of articles with semantically related groups of terms called categories. Category searches allow users to sample a broad range of search terms without having to perform individual searches on each one, and provide a level of search specificity not achievable with simple keyword searches.

**Fig. 7:**
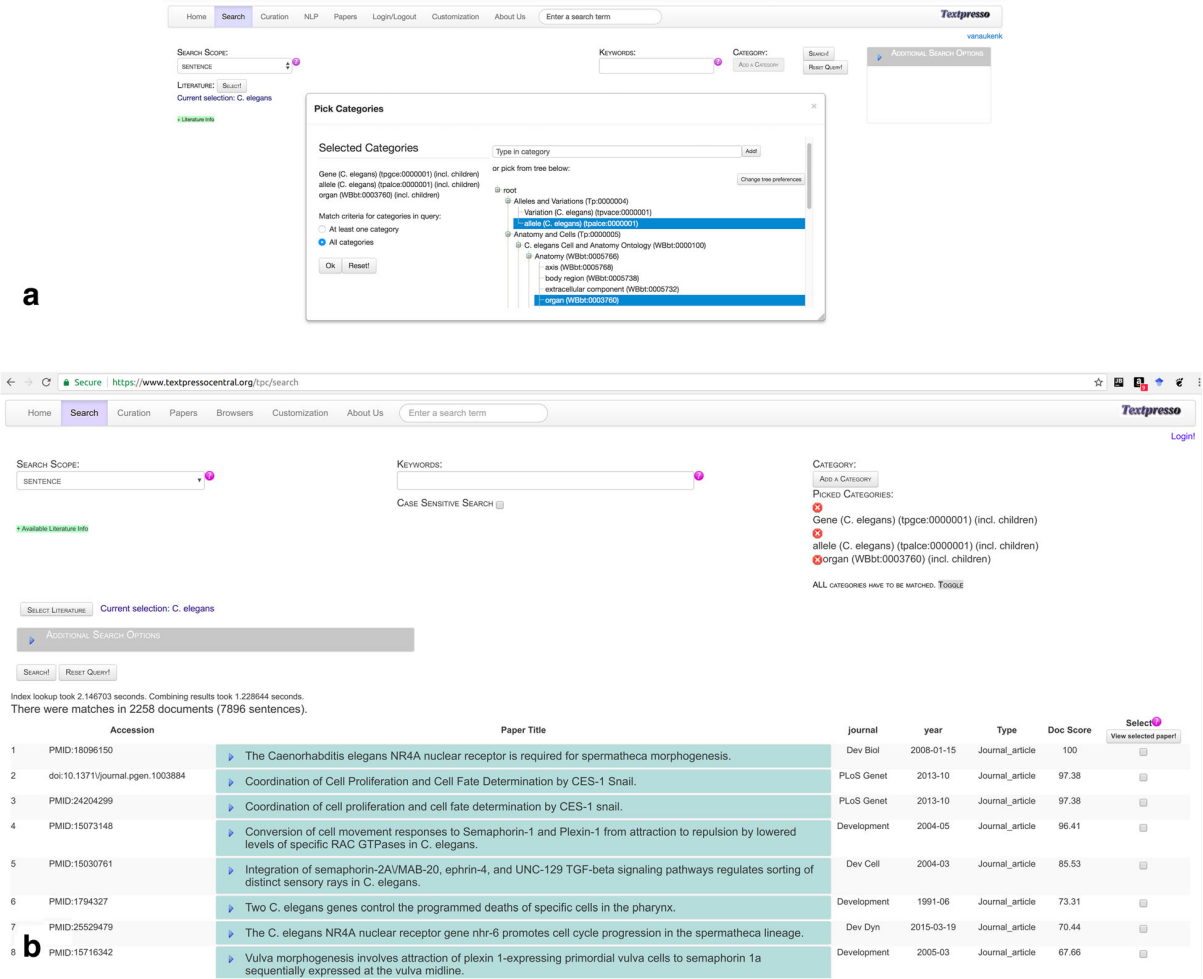
Textpresso Central Category Search. **a** Selecting multiple categories. **b** Search results for the multi-category search of *C. elegans* Genes, *C. elegans* alleles, and *C. elegans* organs

In Textpresso Central, category searches are available from the *Search* module. The workflow for performing a category search is shown in Fig. 7. In this example, the search is tasked with identifying sentences in the *C. elegans* sub-corpus that cite alleles of *C. elegans* genes along with mention of anatomical organs. This type of search might be useful for allele-phenotype curation, a common type of data curated at MODs. From the Search page, the user clicks on the ‘Add a Category’ link. From there, a pop-up window appears that prompts users to either begin typing a category name, or to select categories from the category browser. Three categories are selected for this search: allele (*C. elegans*) (tpalce:0000001); Gene (*C. elegans*) (tpgce:0000001); and organ (WBbt:0003760). For this search, the option to search child terms in each of the categories is also selected and we require that the sentence match at least one term from all three of the selected categories. 7896 sentences in 2258 documents (search performed 2017–11-17) are returned, with papers and sentences again sorted according to score, and matching category terms color-coded according to each of the three selected categories.

**Fig. 8:**
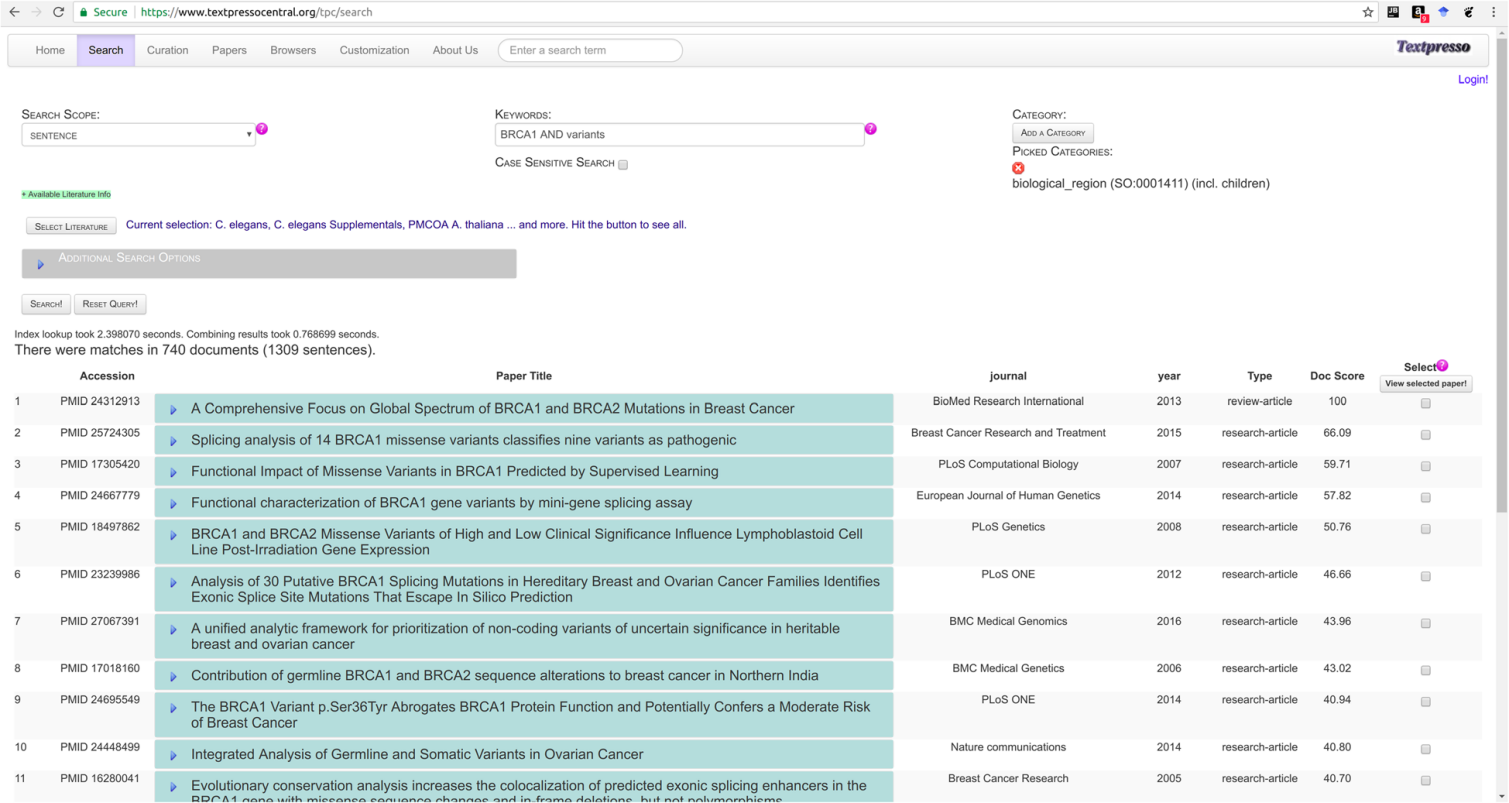
Results of a Textpresso Central Keyword and Category Search

3) Combined keyword and category searches
  Particularly powerful Textpresso Central searches can be performed using a combination of keywords and categories. Figure 8 shows the results of a combined keyword and category search of the entire Textpresso Central corpus that combines two keywords (BRCA1 AND variants) with the SO category biological_region (SO:0001411), a child category of the sequence feature category ‘region’. This search is designed to identify sentences that discuss specific regions of the BRCA1 locus that are affected by sequence variants. This full text search returns 1309 sentences in 740 documents (search performed on 2017–11-17).

### Viewing search results in the context of full text

One of the major advancements in Textpresso Central is the ability to view search results in the context of the full text of the paper. Full text viewing is available for PMC OA articles and articles to which the user, having logged in, has access via institutional or individual subscription. To view search results in the context of the full text, users click on the check box to the right of the Doc Score and then click on the link to ‘View Selected Paper’. To readily find matching returned sentences, highlighted in yellow, users can scroll through them using the scroll functionality at the top right of the page. Further application of viewing the search results in full text will be discussed in the curation case study below.

### Annotation and extraction of biological information using Textpresso central and customized curation forms

As Textpresso searches can make the process of extracting biological information more efficient [7, 13], we sought to improve upon the original system by addressing two of its limitations, namely that curators are best able to annotate when search results are presented within the context of the full text, including supporting figures and tables, and that curation forms, designed by curators in a way that best suits the individual needs of their respective annotation groups, should be tightly integrated with the display of those results.

**Fig. 9:**
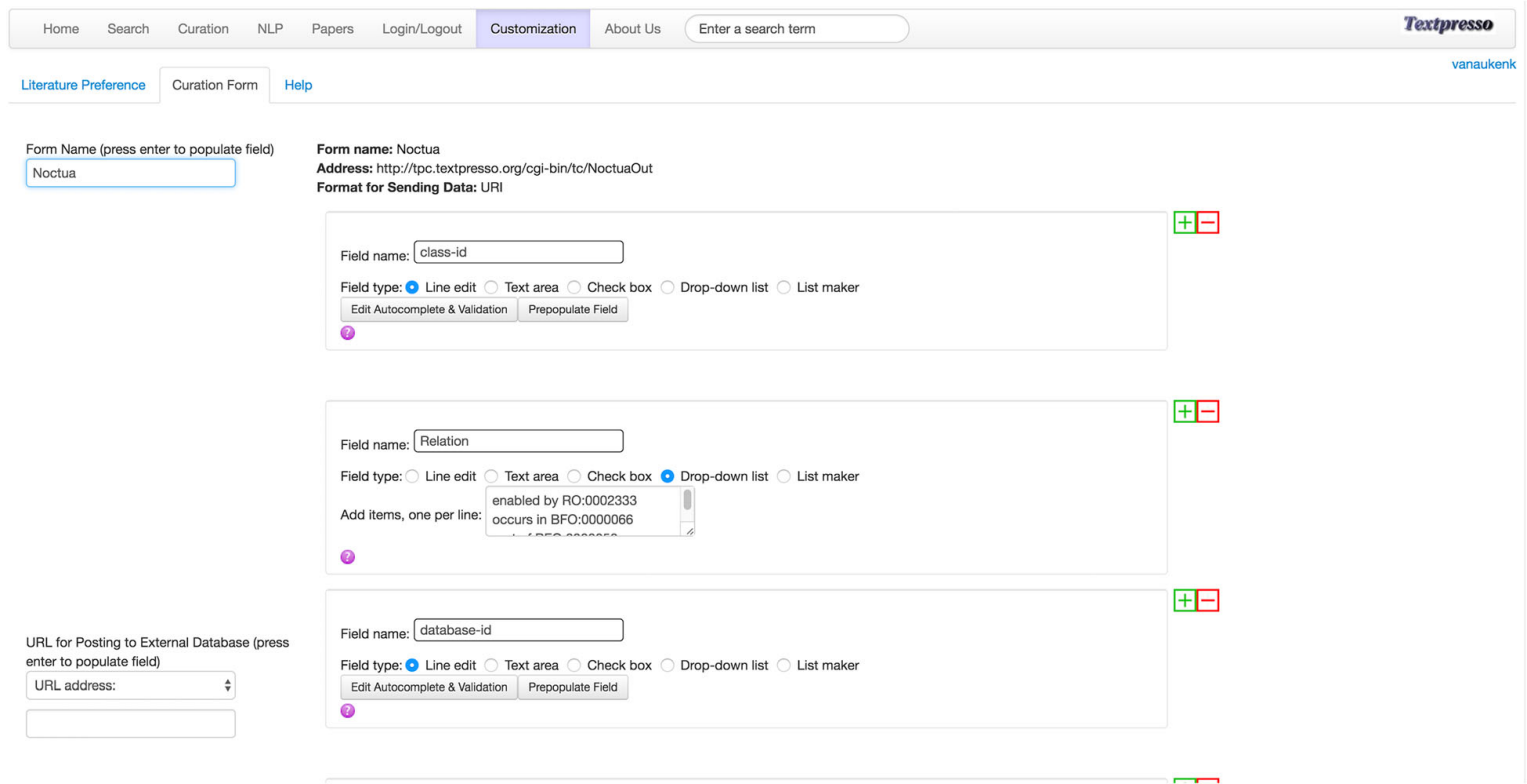
The Textpresso Central Customization Module for Creating Curation Forms

As described in the Methods, customized curation forms can be created by clicking on the *Customization* tab and then the Curation Form tab in the resulting menu. As shown in Fig. 9, once curators have named their form, they are able to add all necessary curation fields, specify population behavior (e.g. autocomplete vs drop-down menu vs pre-population), the format for sending data (JSON or URI), and the location to which all resulting annotations should be sent (URL address). Below, we discuss a specific curation use case using Textpresso Central and the GO’s Noctua annotation tool [48], a web-based curation tool for collaborative editing of models of biological processes built from GO annotations.

### Curation case study: Gene ontology curation

The benefits of Textpresso Central for information extraction and annotation can be illustrated with the following curation case study. GO curation involves annotating genes to one of three ontologies that describe the essential aspects of gene function: 1) the Biological Processes (BP) in which a gene is involved, 2) the Molecular Functions (MF) that a gene enables, and 3) the Cellular Component (CC) in which the MF occurs.

We have previously demonstrated the use of Textpresso to aid in GO CC curation at WormBase [13]. However, we wanted to expand these efforts and integrate Textpresso Central more generally into GO curation pipelines by coupling full text searches with annotation using the GO’s recently developed Noctua curation tool. The Noctua tool can be used to annotate genes using all three GO ontologies. As an example, we will demonstrate how a curator could use Textpresso Central to search the literature for evidence supporting an MF annotation and then send the resulting annotation to Noctua.

Centriole duplication is a key part of the mitotic cell cycle. In *C. elegans*, centriole duplication is regulated, in part, by the *zyg-1* gene which encodes a protein with sequence similarity to protein kinases [49]. To annotate *zyg-1* function, the curator would be interested in identifying experimental evidence for ZYG-1’s protein kinase activity. To begin, the curator first logs into the Noctua annotation tool, navigates to the Paper Markup Tools section of the Edit annotations feature in the Models menu, and then clicks on the Textpresso Central (TPC) link. Clicking on this link directs curators to the Textpresso Central homepage, where they can login and perform the relevant search; in this case, the search is limited to the *C. elegans* corpus and consists of the keyword ‘zyg-1’, and the categories ‘Enzymatic Activity’ and ‘tables and figures’ and their child terms. The latter category is included to restrict matching sentences to those that reference a table or figure in the associated paper. This search returns 45 sentences in 19 documents (search performed 2017–11-17). By reviewing the resulting paper titles and sentences, the curator can select papers for viewing, and possible annotation, in the paper viewer. For this example, we have selected the paper entitled, ‘Phosphorylation of SAS-6 by ZYG-1 is critical for centriole formation in *C. elegans* embryos’ [50].

**Fig. 10:**
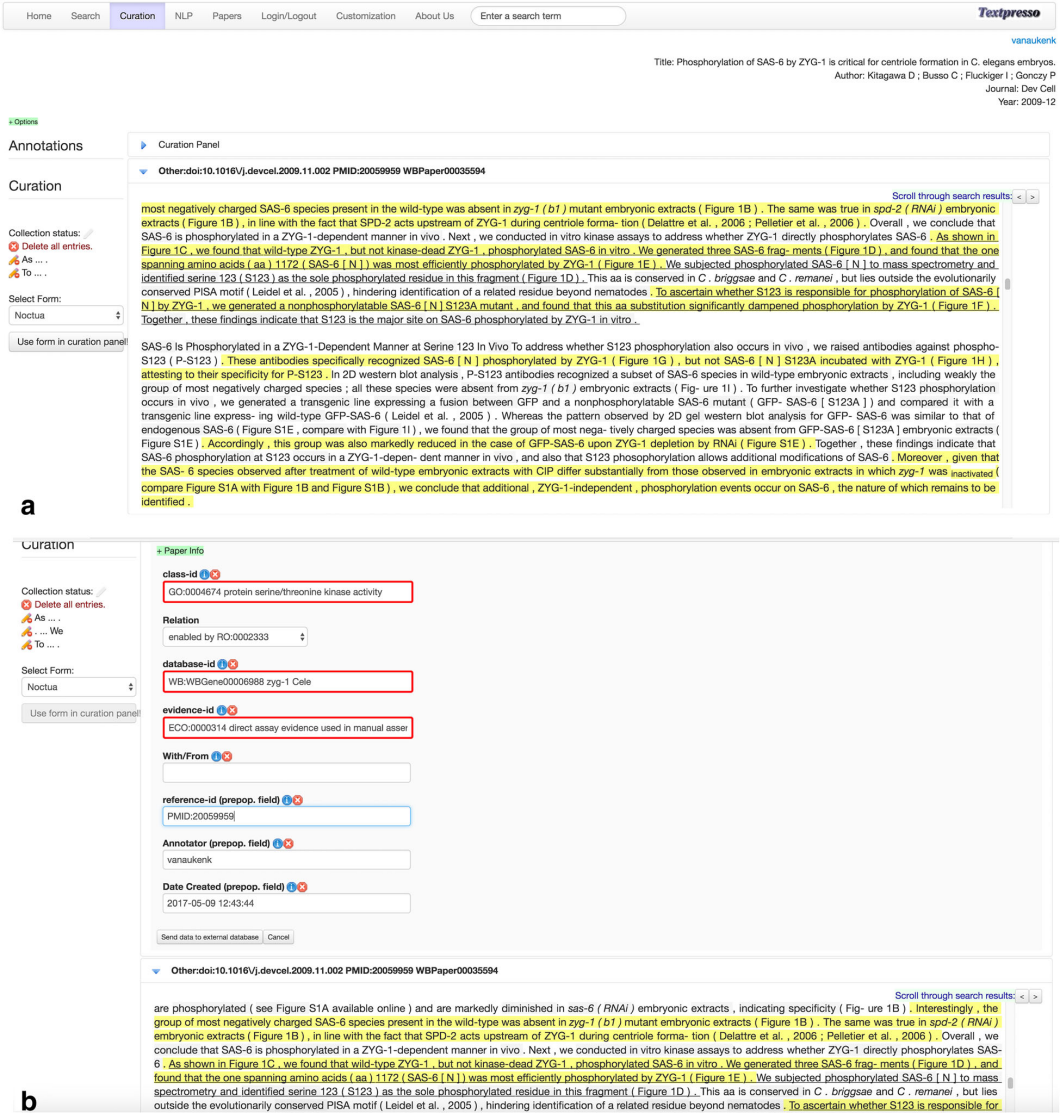
Performing Annotation in Textpresso Central **a** Highlighting Evidence Sentences for Annotation in the Paper Viewer. **b** Creating GO Molecular Function Annotations

**Fig. 11:**
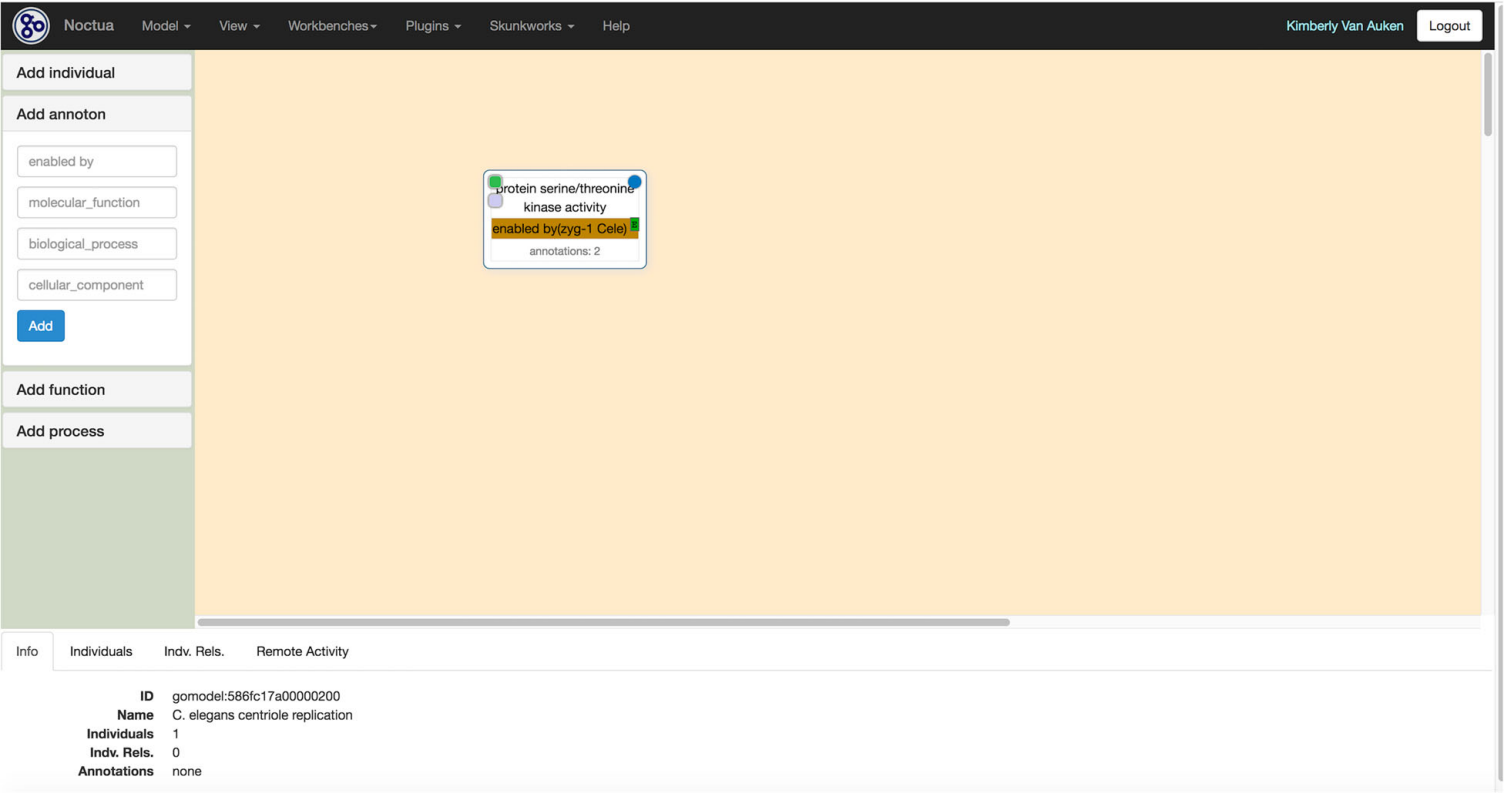
Textpresso Central Annotation Exported to Noctua

Figure 10 shows the selected paper in the Paper Viewer with matching sentences highlighted in yellow. Underlined sentences indicate the evidence statements for ZYG-1 protein serine kinase activity that support annotation to the GO MF term, ‘protein serine/threonine kinase activity’ (GO:0004674). Note that the supporting evidence sentences are non-contiguous which allows curators to collect evidence statements throughout a paper, if needed. Selecting the Noctua curation form brings the curator to the customized curation form, specifically designed by a curator in the curation form editor in TPC to interface between Textpresso Central and Noctua. From the Paper Info widget, the curator can see the selected sentences, their positions in the paper, any additional Textpresso Central annotations, and metadata associated with the annotation, such as the curator name and date. Fields populated via autocomplete using the GO database are shown outlined in red, while pre-populated fields, such as reference-id, Annotator, and Date created are shown in the lower three fields. The Relation field is a drop-down menu, and the curator has selected the appropriate relation from the Relations Ontology [51] for a GO MF annotation in Noctua. Once all necessary field values have been entered, the curator can click on the link to ‘Send data to external database’, click on ‘HTTP Get’ in the resulting window, and the annotation is sent through a parameterized URL to the Noctua curation form with appropriate supporting evidence (Fig. 11). An identifying token originally sent by Noctua (when initially going to the Textpresso Central site from Noctua) is returned to Noctua with the annotation to make sure that the annotation finds its correct place in Noctua’s database. In general, as long as the API of the external database is in the form of parameterized URIs or posts in JSON format, there is no additional configuration necessary on the Textpresso Central site.

## Conclusion

We have developed a system, Textpresso Central, that enables a user to search and annotate a scientific publication in depth, and send curated information to any database in the world. The design satisfies the need for a comprehensive literature search and annotation platform with customized features for optimal use. Textpresso Central is UIMA compliant, making it possible to incorporate external NLP modules, and employs state-of-the-art indexing and web-authoring libraries. Literatures and categories for markup are imported by widely used file formats such as PDF, NXML, and OBO. Furthermore, we have demonstrated the utility of the system through example searches and a real-world curation case study to illustrate how Textpresso Central facilitates biological database curation.

### Future work

While the current system provides a valuable new tool for biocuration, additional features will add to its utility. For example, further development will focus on allowing users to upload and edit their own categories, for paper markup, in a *Category/Lexicon* module. Papers that have been uploaded and organized into literatures will be managed in the *Workflow* module which enables the user to define which papers and corpora should undergo indexing, category markup, and TM and NLP processing (including incorporation of external TM and NLP modules), as well as indexing. We will set up robust TM and ML modules that have proven to be useful, among them a Support Vector Machine module that has been used successfully to classify papers according to the presence of over 10 different data types [52]. We have other ML software packages for models such as CRFs and HMMs, but still need to set them up in such a way that they can be robustly applied to text mining problems specific to the biomedical literature. All modules will be used to implement the computational annotation processing step that has been described in the basic processing pipelines above. There is also a need for handling and managing text spans for the purpose of training TM and NLP modules. To facilitate this, we will develop a *Sentence* module, in which text spans can be collected, viewed, edited, and assigned to specific groups for training and testing purposes. In addition, we will be inviting curators from outside WormBase, as well as users outside the biocuration community, to evaluate TPC and will develop a brief user survey to systematically record their feedback.

We currently subdivide the corpus into more than 25 sub-corpora according to commonly accepted subjects such as Medicine, Disease, Genomics, Genetics and Biology. This subdivision is not set in stone; for example, literatures of particular model organisms will be of interest to the community, too. We will expand the ways of partitioning the literature according to the demands of the community, and also change the way we classify a paper as belonging to one or more sub-literatures, moving away from regular expression matching in title, subject line and journal name to a more comprehensive classifier such as SVMs. Also, the current subdivision that exists in the current system is more of a demonstration model, as meaningful sub-literatures will be established according to requests of the user community. Subdivisions according to organisms, journals or other criteria will be implemented, as needed. We will also consider analyzing MeSH terms that are provided for each article to support new classification schemas.

We would also like to explore making annotations available in BioC format [53]. This format allows sharing text documents and annotations including sentences, tokens, parts of speech, named entities, such as genes or diseases, and relationships between named entities, and thus will enhance the interoperability of Textpresso Central with other systems.

Another addition to the system is a *Paper Browser* module in which frequently used keywords and category terms will be presented in a graphical display relating them. Thus, when the user is mousing over the nodes of the graph, a list of the most relevant papers concerning the corresponding keyword or category term will pop up. The user can then store these papers in lists for further processing or viewing. Finally, we would also like to be able to accept papers in HTML format and not only PDF and NXML format. We will develop a corresponding converter module for this task.

Textpresso Central, like Textpresso, provides full text search functionality. While access to, and processing of, full text may have challenges, e.g. the increased complexity of sentence structure in full text and difficulty in parsing information in figures and tables [54, 55], numerous studies show the need for, and effectiveness of, full text searches over those solely of abstracts [13, 55–58]. Since TPC was developed largely for curators, who require full text to make high-quality annotations, we believe the benefits of providing full text outweigh the challenges, and that by keeping abreast of advancements in article processing and NLP algorithms, we can continue to improve the quality of full text searches.

So far we are using PMCOA as the source of our corpora, and we will include a system that tracks licensing for PDF-based corpora that we will offer, i.e., only PDFs for journals for which a user has a subscription can be used in our system. If there is widespread interest in the community we would seek to acquire licenses for PMC content that is not covered under any open access policies.

All of these elements aid the curator in setting up personalized literature corpora, ontologies, and processing pipelines. Such customizability is an important feature as the adoption of text mining into the biocuration workflow can be difficult owing to potential changes in established workflows and pipelines. Tools like Textpresso Central that help to streamline the curation process by readily coupling state-of-the-art TM and NLP approaches with existing curation databases and workflows have the potential to aid significantly in biocuration. Further, by serving as a means to track the provenance of biological knowledge, Textpresso Central provides a valuable resource for training biocurators, as well as the scientific community, in the methods of literature curation.

## Abbreviations

AE: Analysis Engine
BP: Biological Process
CAS: Common Analysis Structure
CC: Cellular Component
ChEBI: Chemical Entities of Biological Interest
CPE: Collection Processing Engines
CRF: Conditional Random Field
DNA: DeoxyriboNucleic Acid
GO: Gene Ontology
GUI: Graphical User Interface
HMM: Hidden Markov Models
JSON: Javascript Object Notation
MF: Molecular Function
ML: Machine Learning
MOD: Model Organism Databases
NER: Named Entity Recognizers
NLM: National Library of Medicine
NLP: Natural Language Processing
NXML: NLM XML
OBO: Open Biomedical Ontology
PATO: Phenotype and Trait Ontology
PDF: Portable Document Format
PMC: PubMed Central
PMCOA: PubMed Central Open Access
PMID: PubMed identifier
POS: Part-of-Speech
PRO: Protein Ontology
SO: Sequence Ontology
SofA: Subject of Analysis
SVM: Support Vector Machine,
tf*idf: term-frequency*inverse-document-frequency
TM: Text Mining
TPC: Textpresso Central
UIMA: Unstructured Information Management Architecture
URI: Uniform Resource Identifier
URL: Uniform Resource Locator
XML: Extensible Markup Language
